# Synergy of Combined tPA-Edaravone Therapy in Experimental Thrombotic Stroke

**DOI:** 10.1371/journal.pone.0098807

**Published:** 2014-06-09

**Authors:** Yu-Yo Sun, Yury M. Morozov, Dianer Yang, Yikun Li, R. Scott Dunn, Pasko Rakic, Pak H. Chan, Koji Abe, Diana M. Lindquist, Chia-Yi Kuan

**Affiliations:** 1 Department of Pediatrics, Emory University School of Medicine and Children's Healthcare of Atlanta, Atlanta, Georgia, United States of America; 2 Department of Neurobiology, Yale University School of Medicine, New Haven, Connecticut, United States of America; 3 Imaging Research Center, Department of Radiology, Cincinnati Children's Hospital Medical Center, Cincinnati, Ohio, United States of America; 4 Department of Neurosurgery, Stanford University School of Medicine, Stanford, California, United States of America; 5 Department of Neurology, Graduate School of Medicine, Dentistry and Pharmaceutical Sciences, Okayama University, Okayama, Japan; University of South Florida, United States of America

## Abstract

Edaravone, a potent antioxidant, may improve thrombolytic therapy because it benefits ischemic stroke patients on its own and mitigates adverse effects of tissue plasminogen activator (tPA) in preclinical models. However, whether the combined tPA-edaravone therapy is more effective in reducing infarct size than singular treatment is uncertain. Here we investigated this issue using a transient hypoxia-ischemia (tHI)-induced thrombotic stroke model, in which adult C57BL/6 mice were subjected to reversible ligation of the unilateral common carotid artery plus inhalation of 7.5% oxygen for 30 min. While unilateral occlusion of the common carotid artery suppressed cerebral blood flow transiently, the addition of hypoxia triggered reperfusion deficits, endogenous thrombosis, and attenuated tPA activity, leading up to infarction. We compared the outcomes of vehicle-controls, edaravone treatment, tPA treatment at 0.5, 1, or 4 h post-tHI, and combined tPA-edaravone therapies with mortality rate and infarct size as the primary end-points. The best treatment was further compared with vehicle-controls in behavioral, biochemical, and diffusion tensor imaging (DTI) analyses. We found that application of tPA at 0.5 or 1 h – but not at 4 h post-tHI – significantly decreased infarct size and showed synergistic (p<0.05) or additive benefits with the adjuvant edaravone treatment, respectively. The acute tPA-edaravone treatment conferred >50% reduction of mortality, ∼80% decline in infarct size, and strong white-matter protection. It also improved vascular reperfusion and decreased oxidative stress, inflammatory cytokines, and matrix metalloproteinase activities. In conclusion, edaravone synergizes with acute tPA treatment in experimental thrombotic stroke, suggesting that clinical application of the combined tPA-edaravone therapy merits investigation.

## Introduction

Thrombolysis is the most effective therapy of acute ischemic stroke, but recanalization of large arteries does not secure reperfusion of the distal vascular bed, which is a better predictor of clinical outcomes [1], [2]. The mechanism of reperfusion deficits despite large-artery recanalization are complex, including breakup of the primary clot to obstruct smaller arteries, compression of blood vessels by brain edema, and secondary thrombosis in the peri-infarct area [3]. Combining thrombolysis with antioxidants may improve vascular reperfusion and clinical outcomes, but with the negative clinical trial of combined tPA and NXY-059 therapy in ischemic stroke, there have been few attempts to revive the thrombolysis-antioxidants strategy in the United States [4].

It is important that new thrombolytic therapies should be evaluated in thromboembolic models before clinical trials [5]. The intraluminal suture middle cerebral artery (MCA) occlusion model is less useful for this purpose because it induces blood clots inefficiently. Further, vascular reperfusion after removal of the suture is much faster than the response to thrombolysis in stroke patients, resulting in a long therapeutic window that may not be realistic [6]. However, current thromboembolic models also have limitations, because they either require craniectomy to inject thrombin directly into the MCA or rely on introduction of pre-formed emboli through the carotid artery, which may cause large variations in location and infarct size [7], [8].

Given this context, we have two objectives in the present study.

First, we wanted to test whether transient ligation of the unilateral common carotid artery plus hypoxia will induce thrombosis and infarction. We have shown that permanent ligation of the unilateral common carotid artery plus hypoxia triggers blood clotting and infarction [9]. If transient hypoxia-ischemia (tHI) has similar effects and responds favorably to tPA thrombolysis, it may provide a simple and more standardized thrombotic stroke model.

Second, we asked whether edaravone, a free radical scavenger that has been approved in Japan for treating ischemic stroke within 24 h of onset, enhances tPA-thrombolysis in this novel stroke model. As a neuroprotectant, edaravone has several advantages over NXY-059, including greater permeability across the blood-brain-barrier [10]. It also ameliorates thrombolysis-related hemorrhage in preclinical models, but whether combined tPA-edaravone therapy is more potent in reducing infarct size is unclear [11], [12]. In fact, they failed to show additive benefits in a rabbit small clot embolic model, likely due to a ceiling effect of high-dose tPA [13]. Because the most likely (and ethical) clinical trial design in ischemic stroke will be comparing the outcomes of patients receiving either tPA or combined tPA-edaravone therapy, it is critical to first examine the safety and benefits of similar treatments in preclinical models.

Our results showed that tHI insults reliably induce thrombosis and cerebral infarction, and responds favorably to acute tPA-thrombolysis plus synergistic effects with the edaravone treatment. These results support potential benefits of combined tPA-edaravone therapy in acute ischemic stroke.

## Materials and Methods

### Stroke surgery

Male C57BL/6 mice and Thy1-YFP mice (Jackson Laboratories, Bar Harbor, ME) at the age of 10 to 13 weeks were subjected to transient cerebral HI, performed with minor modifications as previously described [9], [14]. Animals weighing 22–30 g were anesthetized under 2% isofluorane to perform transient occlusion of the right common carotid artery (tCCAo) with two releasable knots of 4.0 silk suture, which were released after the hypoxic stress. For inducing hypoxia, mice were infused with 7.5% O_2_/92.5% N_2_ through a face-mask for 30 min. Throughout surgery and tHI insult, the core body temperature of animals was maintained at 37.5+/−0.5°C using a rectal thermoprobe (EW-89000-10; Cole Parmer, Vernon Hills, IL) coupled to a heating lamp. After hypoxic exposure, mice were returned to dams in the animal care facility. Neonatal HI procedure was performed in seven-day-old rats, as previously described [15]. Animals were randomized for treatments and the investigators analyzing brain infarction were blinded to the treatments. All animal procedures were approved by the Institutional Animal Care and Use Committee (IACUC) of Emory University and conform to the National Institutes of Health Guide for Care and Use of Laboratory Animals, as well as the ARRIVE guidelines (Animals in Research: Reporting *In-Vitro* Experiments).

### Drug administration

All animals (total n = 166 for the primary end-point study) were randomly divided into 8 groups with at least 10 mice each. Group 1 is the vehicle-treatment group with tail vein injection of the solvent used for recombinant tPA (Activase). Group 2 receive the edaravone treatment that consists of intraperitoneal injection of 3 mg/kg edaravone (a generous gift of the Mitsubishi Tanabe Pharma Cooperation, Osaka, Japan) at 0, 1 and 2 h after tHI. Groups 3–5 receive tail vein injection of 10 mg/kg recombinant tPA (Activase, Genetech, USA) at 0.5, 1, or 4 h following the tHI insult. Groups 6–8 receive the combined therapy with edaravone (at 0, 1, and 2 h post-tHI) and tPA (at 0.5 or 1 or 4 h post-tHI).

### Post-surgery monitoring and measurement of brain infarction

Post-stroke surgery mice were closely monitored for 2 h and then every 6 h until 24 h recovery. Animals showing sign of pain or distress were treated with analgesics (Ketoprofen, 3–5 mg/kg every 24 h subcutaneously, or Meloxicam, 2 mg/kg every 12–24 h by oral gavage). Mice treated with analgesics were excluded from biochemical analysis, as these medicines may interfere with pathological responses. Animals in intractable distress or a moribund state were humanly euthanized by asphyxiation with carbon dioxide. Detection of infarction was performed by in-vivo TTC staining at 24 h after surgery by a lab member unaware of the animal treatment. The brains were further fixed with 4% paraformaldehyde overnight and sectioned into 0.7 mm thickness by Vibratome (Stoelting, Wood Dale, IL). Brain slice images (4 slices) were analyzed using the ImageJ 1.4 software (NIH, Bethesda, MD) and quantified as the ratio of the infarcted area in the ipsilateral hemisphere to the total area of the uninjured, contralateral hemisphere as previously described [16].

### Laser Speckle Contrast Imaging

Cerebral blood flow (CBF) was measured using a two-dimensional laser speckle contrast imaging system [17], and following the manufacturer's instruction (MoorFLPI-2; Moor Instruments Inc. Delaware, USA). Briefly, anesthetized mice were placed in the prone position with the skull exposed but unopened. The CBF were measured in both cerebral hemispheres and recorded immediately after the CCAo surgery, during hypoxia (30 min) and reperfusion (20 min). Also shown are CBF images at the selected time-points from the entire recording. CBF was analyzed by the MoorFLPI software and shown with arbitrary units in a 16-color palette.

### tPA/uPA and MMP-9 activities

The plasminogen activator (tPA and uPA) and matrix metalloproteinase-2 and 9 (MMP-2/9) activities were measured by in-gel substrate zymography as described previously [15], [18]. The tPA/uPA and MMP-2/9 activities were normalized by the immunoblot against β-actin in brain extracts or transferrin in serum, respectively. The tPA activity in brain extracts was also measured with a fluorescent kit as previously described [18].

### Immunohistochemistry and Immunoblot analysis

Immunohistochemistry and immunoblot were performed at 1 or 4 h after stroke and detected as previously described [16]. The following antibodies for immunohistochemistry were used: rabbit anti-fibrinogen (a gift of Dr. J. Degen), rat anti-glycoprotein IIb (CD41, BD, San Diego, CA), and rat anti-P-Selectin (BD). The following antibodies were used for immunoblot: rabbit anti-tPA (Molecular Innovations, Novi, MI), mouse anti-PAI-1 (Molecular Innovations), and mouse anti- β-actin (Sigma, St. Louis, MO).

### Histological assay of vascular obstruction

Evans blue albumin was used to evaluate vascular perfusion and BBB leakage as previously described [19]. Briefly, mice received tail vein injection of 200 μl 2% Evans Blue dye solution (Sigma) at 2 or 4 h after tHI. After 10 min of Evans blue circulation, mice were killed and the brains were quickly removed into 4% paraformaldehyde. Fixed brains were sectioned at 100 μm thickness and the fluorescence was observed using a 680 nm emission filter on a fluorescent microscope.

### Real time reverse-transcription PCR

Real time RT-PCR was performed at 24 h recovery and detected with the SYBR green dye as previously described [20]. The following primers were used.

*Tspo*: 5′-CTATGGTTCCCTTGGGTCTCTAC-3′, 5′-AGGCCAGGTAAGGATACAGCAAG-3′ *IL-6*: 5′-GGAGAGGAGACTTCACAGAGGAT-3′, 5′-AGTGCATCATCGCTGTTCATAC-3′

*IL-1β*: 5′-CTTTCGACAGTGAGGAGAATGAC-3′, 5′-CAAGACATAGGTAGCTGCCACAG-3′

*Gapdh*: 5′-CTCATGACCACAGTCCATGC-3′, 5′-TTCAGCTCTGGGATGACCTT-3′

### Detection of oxidative stress

Lipid peroxidation was measured by quantifying malondialdehyde (MDA) in brain extracts at 24 h recovery using a commercial kit (OxiSelect; Cell Biolabs Inc., San Diego, CA) as previously described [14]. The superoxide production was determined by *in situ* oxidized hydroethidine detection (HEt, Invitrogen, Carlsbad, CA) as previously described [21]. The HEt solution (1 mg/ml) was injected through the tail vein to the animals at 30 min before sacrifice. Oxidized HEt was detected at an emission of 590 nm and quantified the ratio to DAPI-positive nuclei in four randomly selected visual fields (100X) in the ipsilateral cerebral cortex for comparison.

### Long-term outcome and neurological deficit analysis

Long-term outcome was evaluated by the survival rate and neurological deficits over 7 days after stroke surgery. Neurological deficits were scored using a 6-point system (0–5) as described [9]. Mice with no apparent deficit were scored as 0; 1 indicates the failure of forelimb extension; 2 denotes normal posture at rest but walking in large circles to the contralateral side; 3 refers to walking in small circles and/or rolling over to the contralateral side repeatedly; 4 is lying nearly motionless on the contralateral side; 5 means death. Rotarod testing was examined at 7 d after surgery as previously described [18]. Mice were placed on an accelerating rotating rod from 4 to 40 rpm over 1 min and their latency to fall (seconds) was recorded.

### Diffusion tensor imaging (DTI)

Due to the lower signal-to-noise ratio and lower resolution of in vivo DTI in small rodent brains, ex vivo imaging was performed to assess WM damage at 24 h recovery after various treatments as previously described [14], [18]. The brains were equilibrated to the ambient bore temperature for ∼30 min. All ex vivo images were acquired with a custom-built solenoid transmit/receive coil. The DTI parameters were: b = 800 seconds/mm^2^, gradient separation/duration (D/δ)  = 12/4 msec, FOV = 32×12×12 mm3, TE/TR = 22/1,000 msec, 1 average, matrix  = 256×96×96, resolution  = 125 μm isotropic. The total scan time was ∼18 hrs. Quantification of DTI measures including FA, radial and axial diffusivity was performed using DTIstudio software.

### Electron microscopy (EM)

EM analysis of the ultrastructural pathology of white matter was performed after the DTI scan as previously described [14]. Briefly, animals were perfused cardially with 4% paraformaldehyde and 1% glutaraldehyde in 0.1 M phosphate buffer prior to the DTI scan. After the DTI scan, the brains were sectioned into 100-μm-thick slices by a vibratome. The sections were postfixed with 1% OsO_4_, stained with 2% uranil acetate during dehydration and embedded in Durcupan (ACM; Fluka, Buchs, Switzerland) on a microscope slide and cover-slipped. Fragments of studied axonal tracts were re-embedded into Durcupan blocks and cut by a Reichert ultramicrotome into 70-nm-thick sections. The ultrathin sections were then stained with lead citrate and evaluated in a JEOL 1010 electron microscope equipped with a Multiscan 792 digital camera (Gatan, Pleasanton, CA).

### Statistical analysis

Statistical analysis was performed using one-way ANOVA followed by the posttest of Newman-Keuls or unpaired *t*-test for two samples. P values less than 0.05 were considered a significant difference. All values were expressed as mean +/− SEM.

## Results

### Transient hypoxia-ischemia is sufficient to induce reperfusion deficits and infarction

We previously reported that permanent occlusion of the unilateral common carotid artery plus 30–60 min exposure to hypoxia (7.5% oxygen) triggers blood clotting and infarction in adult mice [9]. The previous study also showed that the cerebral blood flow (CBF) recovered poorly after hypoxia even with the removal of carotid artery ligation, suggesting that transient hypoxia-ischemia (tHI) insults may suffice to trigger endogenous thrombosis.

**Figure 1:**
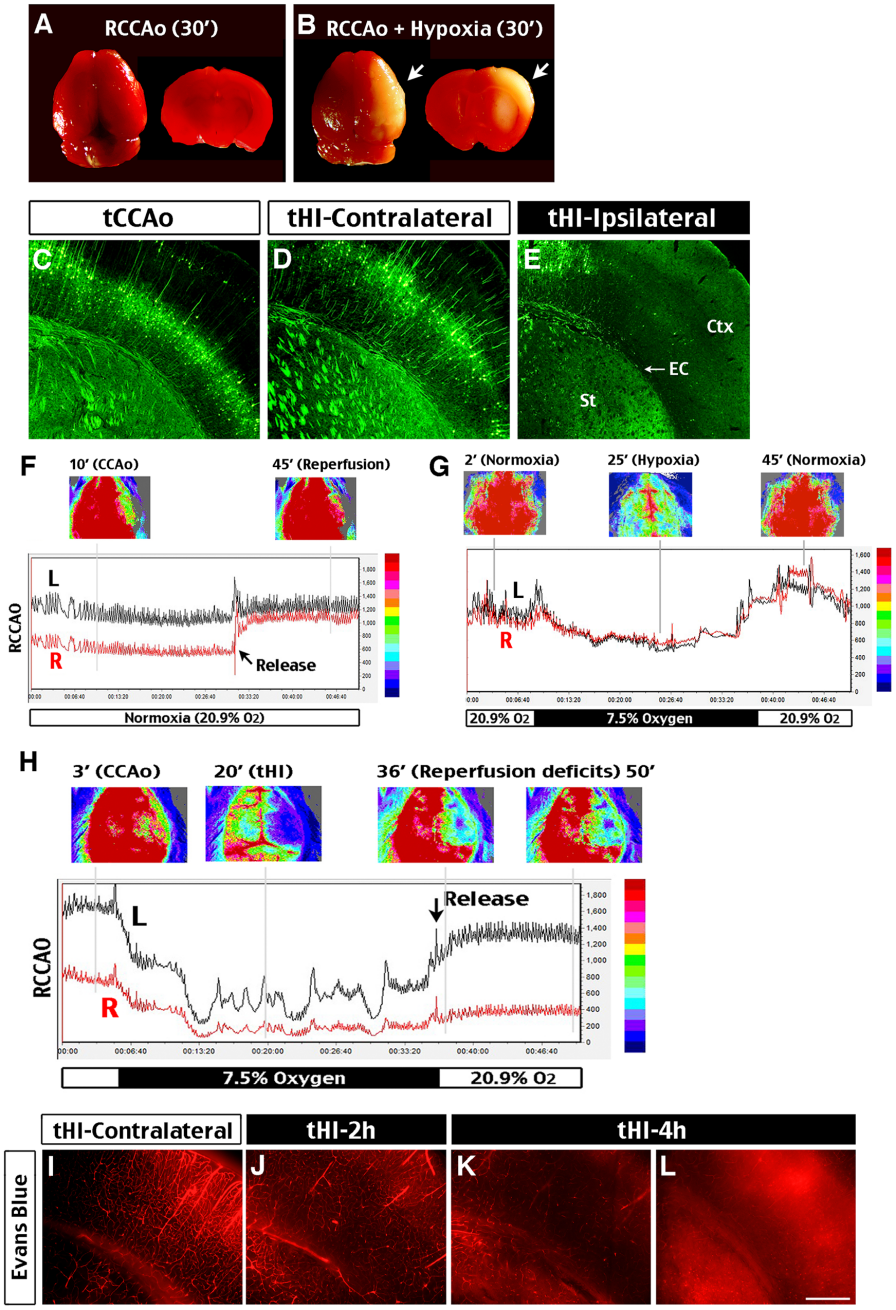
Imparied cerebral vascular reperfusion after transient hypoxia-ischemia (tHI). (**A, B**) TTC-staining detected no visible infarction at 24 h after 30-min transient occlusion of the right common carotid artery (RCCAo), but exposing mice to 7.5% oxygen during the ischemic period (RCCAo + Hypoxia) produced sizeable infarction in the ipsilateral hemisphere. (**C–E**) By subjecting Thy1-YFP mice to 30-min unilateral carotid occlusion (tCCAo) or carotid occlusion with hypoxia (tHI), we detected loss of deep cortical neurons and destruction of white matter in the striatum (St), external capsule (EC), and cerebral cortex (Ctx) in the ipsilateral hemisphere following tHI insult (E), but not on the contralateral hemisphere (D) or after the tCCAo insult. (**F, H**) Laser speckle contrast imaging showed the effects of hypoxia (7.5% oxygen) on cerebral blood flow (CBF) during and after transient unilateral occlusion of the common carotid artery (RCCAo; R indicates the carotid-ligated hemisphere; L, the contralateral hemisphere). When it was under normoxia (F), CBF on the carotid-ligated hemishere was at ∼50% throughout 30-min ischemia, and recovered to >85% within 3 min after the release of vascular occlusion. When it was under hypoxia during carotid occlusion (H), CBF on the ipsilaterla side dropped to <20% of the contralteral value during ischemia-hypoxia, and rarely recovered to above 30% after release of the carotid artery ligation. Shown are representative CBF tracings for n >4 in each group. The 2-dimensional CBF images correspond to the indicated timespoints (also marked by grey lines in the tracing). (**G**) CBF response to 30 min hypoxia (7.5% oxygen) without carotid artery ligation. During hypoxia, CBF dropped to 76% of the baseline value on both hemispheres, and transiently rebounded to ∼130% at the end of hypoxic stress. (**I–L**) Detection of vascular perfusion by tail-vein injection of Evans Blue dye at 10 min before sacrificing the mouse. Evans Blue dye filled vessels in the contralateral hemisphere following tHI insult, but was excluded in pocketed areas in the ipsilateral hemisphere at 2 h, and further restricted or leaked in the brain parenchyma at 4 h (n>3 for each time-point). Scale bar: 250 μm in C–E, H–K.

To test this hypothesis, we compared the responses of adult male Thy1-YFP mice to 30 min ligation of the right common carotid artery in an ambient atmosphere or exposure to 7.5% oxygen. We found that while transient carotid occlusion (tCCAo) produced no discernible brain injury, the tHI insult consistently led to damage in the MCA-territory, as shown by TTC stain at 24 h recovery (Fig. 1A, B). The tHI insult also injured white matter in the striatum and external capsule, but not in the contralateral hemisphere or by the tCCAo insult alone (Fig. 1C–E).

To explore the causes of tHI-induced infarction, we used laser speckle contrast imaging [17] to compare the effects on CBF by 30-min transient carotid occlusion in a normoxic (20.9% oxygen) or hypoxic atmosphere (7.5% oxygen), as well as inhalation of hypoxic air alone (Fig. 1F–H). In transient ischemia under normoxia, the CBF on the carotid-ligated hemisphere was stabilized at ∼50% of the contralateral value and rapidly recovered to >85% after release of the carotid occlusion (Fig. 1F). In inhalation of hypoxic air alone, the CBF declined to ∼75% of the baseline value, and rebounded transiently to ∼130% upon returning to normoxia (Fig. 1G). Yet, in transient ischemia under hypoxia, the CBF on the ipsilateral hemisphere plummeted to <20% of the baseline level in 10 min, and rarely recovered above 30% even at 20 min after the release of carotid artery ligation and returning to the ambient atmosphere (Fig. 1H). In contrast, the CBF in the contralateral hemisphere fluctuated between 20 to 50% during hypoxia, and recovered quickly to above 80% after the tHI insult.

We also used tail vein-injection of the Evans Blue dye to assess cerebral perfusion at later time-points after tHI insults. This analysis showed impaired vascular perfusion when compared to the contralateral side at 2 h after tHI, (Fig. 1I, J). At 4 h post-tHI, we detected larger areas of low-perfusion or extravasation of the Evans Blue dye (Fig. 1K, L). These results indicated that as brief as 30-min tHI insult is sufficient to induce reperfusion deficits and sizable cerebral infarct.

### Transient hypoxia-ischemia induces endogenous thrombosis and suppresses tPA activity

**Figure 2:**
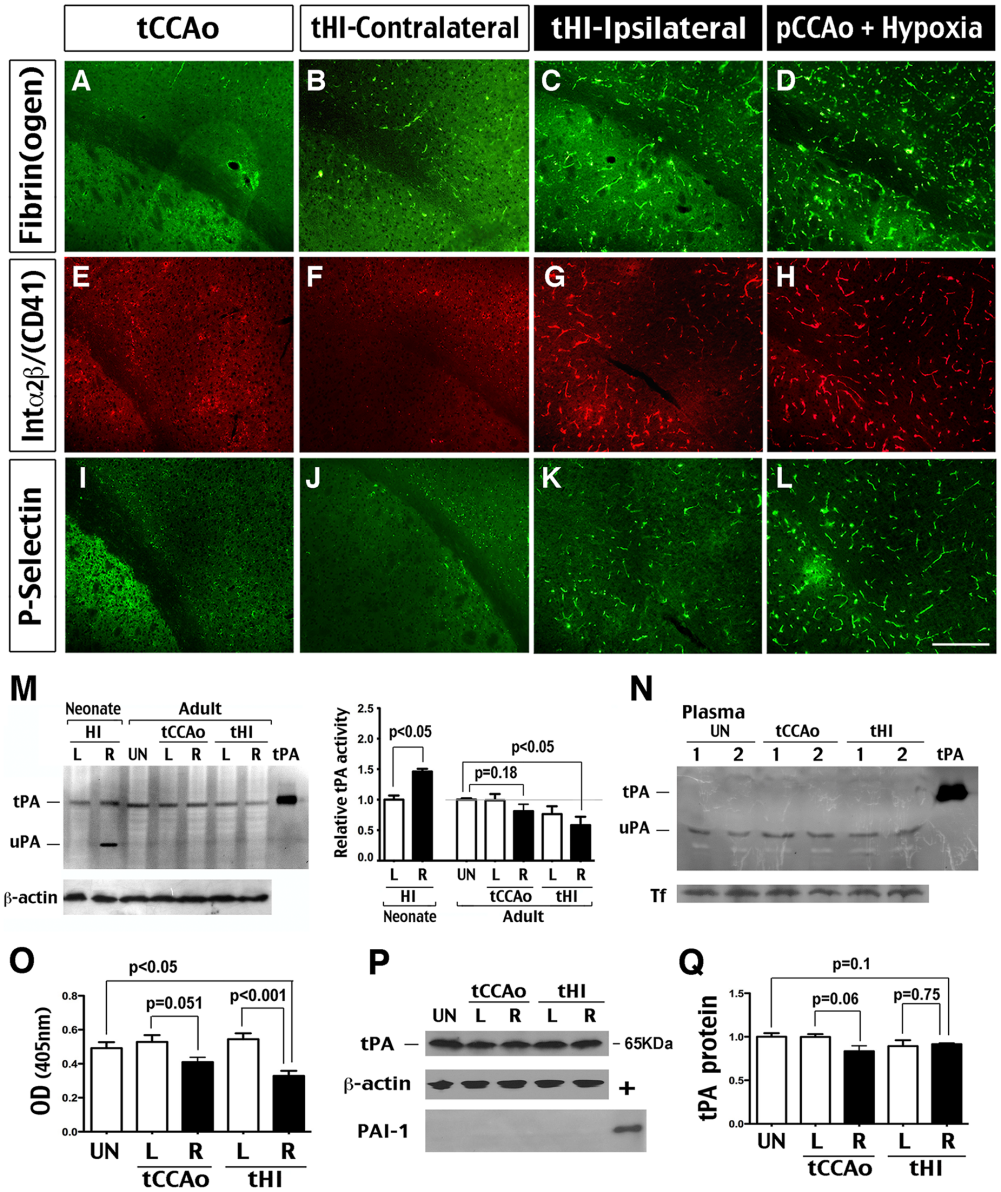
Spontaneous thrombosis and decreased tPA activity following tHI insults. (**A–L**) Immunostaining performed at 1 h after tCCAo, tHI or permanent carotid ligation (pCCAo) plus hypoxia showed deposits of fibrin(ogen) and platelets (detected by anti-Integrin α2β/CD41) and P-Selectin-positive blood vessels (a marker for endothelial activation) and in the ipsilateral hemisphere following tHI insults (C, G, K). Permanent carotid ligation plus hypoxia produced the same staining pattern, but with stronger signals of all three markers (D, H, L). This staining pattern was absent in the brain after tCCAo (A, E, I) or on the contralateral hemisphere following tHI (B, F, J) (n>4 for each). (**M**) Plasminogen-zymogram showed definitive induction of the brain uPA and ∼50% increase of tPA activity in the ipsilateral hemisphere (R) at 4 h post-HI in neonates. No uPA-induction was found in adult brains at 4 h following tCCAo or tHI insults. The tPA activity in the ipsilateral hemisphere (R) following tHI was reduced to ∼67% of the level in unchallenged (UN) brains (p<0.05). The right panel is the quantification of zymogram band intensity (n = 4 for each). (**N**) The plasminogen-zymogram from plasma showed the lack of tPA activity and a similar degree of uPA activity at 4 h after tCCAo or tHI insults in adult mice. Immunoblot detection of transferrin (Tf) served as the internal control. (**O**) Direct measurement of tPA activity using a fluorescent substrate kit also showed significant reduction in the ipsilateral hemisphere at 4 h following tHI insult in adult brains (n = 3-4 for each). (**P–Q**) Immunoblot analysis showed minimal change of the tPA protein level and no sign of plasminogen activator inhibitor-1 (PAI-1) up-regulation after tCCAo or tHI insult in adult brains (n = 4 for each). Scale bar: 200 μm in A–L.

We have shown that permanent unilateral carotid ligation plus hypoxia triggers aggregation of fibrins and platelets (i.e. thrombosis) [9]. To test whether the tHI insult also impairs hemostasis, we perfused mice at 1 h after transient carotid artery ligation (tCCAo), tHI, or permanent carotid artery ligation plus hypoxia (pCCAo + Hypoxia), while keeping the duration of carotid occlusion or hypoxia at 30 min evenly, and examined the brains by immunocytochemistry. This analysis showed that tCCAo yielded negative staining of fibrin(ogen), platelets (labeled by anti-integrin α2β), and P-Selectin (a marker of endothelial activation) (Fig. 2A, E, I). After tHI, a trace amount of platelet-free fibrins, but no immunoreactivity to P-Selectin, was detected in the contralateral hemisphere (Fig. 2B, F, J). In contrast, tHI and pCCAo + Hypoxia insults both led to widespread fibrin deposits, platelet aggregation, and endothelial activation (Fig. 2C, D, G, H, K, L). These results suggested that tHI insults induce endogenous thrombosis in adult brains, which stands in contrast to rapid recovery of CBF following HI insults in rodent neonates [15].

To explain the age-differential responses, we compared the brain parenchymal tPA and urinary-type plasminogen activator (uPA) activities at 4 h after the neonatal HI, adult tCCAo, or adult tHI insult. Zymography showed that neonatal HI insult caused marked induction of uPA and 50% increase of the tPA activity, while adult tHI insult not only failed to induce uPA, but rather decreased the tPA activity (Fig. 2M). In contrast, the tCCAo insult had little effect on tPA or uPA activity in adult brains. We also compared the effects of tCCAo and tHI insults on the plasma plasminogen activators, which showed a very low baseline tPA activity and little change of the uPA activity after either insult in adults (Fig. 2N). These results suggested that tHI insult reduced tPA activity in the vascular wall or brain parenchyma. The adult tHI-mediated reduction of tPA activity was corroborated by a fluorogenic substrate assay (Fig. 2O) [22]. We also showed that HI insults did not alter the protein level of tPA and plasminogen activator inhibitor-1 (PAI-1) in brain parenchyma (Fig. 2P, Q).

### Edaravone and early post-tHI administration of tPA have synergistic benefits

**Figure 3:**
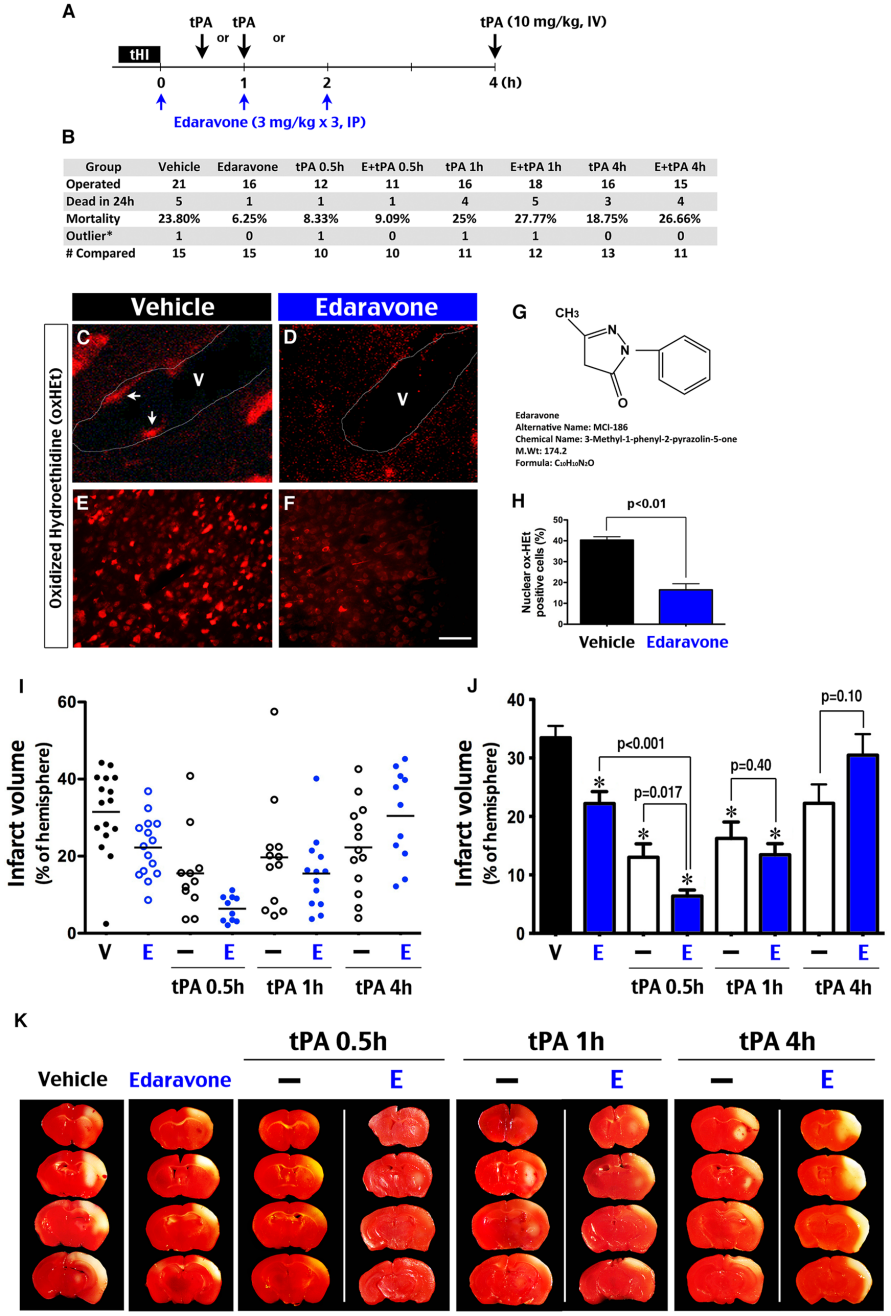
Synergy between edaravone and early tPA-infusion therapies in the tHI model. (**A**) Scheme of experimental treatments tested in this study. tPA (10 mg/kg) was injected through the tail vein at 0.5, 1, or 4 h following the tHI insult. The edaravone treatment consists of 3 doses (3 mg/kg, IP) immediately after, and at 1, and 2 h post-tHI. (**B**) Tabulation of the number of operated animals, dead within 24 h, considered outliers (outside the mean +/−2 SD), and those included for comparison in each treatment group. (**C–F, H**) Detection of superoxide, indicated by the fluorescent hydroethidine (oxHEt), at 2.5 h after tHI. This assay showed numerous oxHEt positive nuclei along the blood vessel wall and in the cerebral cortex of mice that received the vehicle-treatment. The number of oxHEt-positive nuclei was greatly reduced in both locations in edaravone-treated animals. H is the quantification of oxHEt-positive nuclei (over DAPI-staining) in 4 randomly selected visual fields. The p-value was determined by *t*-test. (**G**) Chemical formula, structure and name of edaravone (also called MCI-186). (**I**) The mean and infarct volume of individual animals in each treatment group. (**J**). Statistical analysis of the infarct size in each treatment group (shown are the mean and standard error). The p-values are determined by *t*-test; * p<0.001 compared to the vehicle control group. (**K**) Representative TTC-stained brain slices at 24 h after tHI insult in each treatment group. Pale staining indicates infarcted tissue. Scale bar: 10 μm in C, D; 20 μm in E, F.

The observation of tHI-mediated suppression of tPA activity and infarction suggests that supplement of tPA may reduce cerebral infarct in this stroke model. Furthermore, the addition of edaravone may strengthen thrombolysis in this paradigm, because a recent study suggested that administration of edaravone during tPA infusion enhances early recanalization in stroke patients [23]. To test these hypotheses, we compared the outcomes of tHI-injured mice in eight post-tHI treatment groups, including tail vein injection of tPA (10 mg/kg bolus at 0.5, 1, or 4 h) or the vehicle (0.5 h), intraperitoneal injection of edaravone (3 mg/kg at 0, 1, and 2 h), or edaravone combined with tPA application at 0.5, 1, or 4 h (E + tPA 0.5, 1, or 4 h) (Fig. 3A, B).

In pilot studies, we found that post-tHI application of edaravone, a lipophilic free radical scavenger (Fig. 3G), decreased oxidized hydroethidine (oxHEt, a marker of superoxide anion [21])-positive nuclei in the cerebral blood vessel wall and conferred ∼50% reduction of oxHEt-positive nuclei in the cerebral cortex at 2.5 h recovery (Fig. 3 C–H). These results support the idea that edaravone mitigates oxidative stress in both neurons and vascular endothelial cells [24].

In vehicle-treated animals, the mortality rate at 24 h post-tHI was 23.8% and only one in 21 tHI-injured mice was an outlier (outside the mean +/−2 SD) (Fig. 3B, I). When compared with the embolic stroke model [25], the tHI model caused a similar mortality rate (13–23%) and comparable, if not better, reproducibility of brain damage. By average, the tHI insult caused 31.5% infarct volume in vehicle-treated mice at 24 h recovery, with the lesion primarily located in the MCA-supplied territory, including the striatum and the lateral cerebral cortex (Fig. 3I–K; shown in I are the mean and individual's infarct size; in J, mean +/− standard error of the infarct volume; * indicates p<0.001 compared to vehicle controls with the p-values determined by *t*-test; in K, representative TTC-staining in each treatment at 24 h survival). Of note, the tHI-induced infarction was smaller than those incurred by permanent carotid ligation plus hypoxia [9].

Compared with vehicle-treated mice, early intervention with tPA (0.5 and 1 h) provided significant benefits, which was reduced in late intervention (4 h). When tPA was applied at 0.5 h post-tHI, the mortality rate was reduced from 23.8% (in vehicle-controls) to 8.3% and the mean infarct volume decreased from 32.5% (in vehicle controls) to 15.5% (p<0.001). When tPA was injected at 1 h recovery, the mortality rate was similar to controls (25%), but the infarct size was reduced to 19.7% (p<0.001 compared to the vehicle group). Yet, when tPA was applied at 4 h post-tHI, the mortality rate (18.7%) and infarct volume (22.3%) were only modestly reduced from vehicle-treated mice (Fig. 3I). Of note, delayed tPA-treatment at 4 h in the tHI model did not induce hemorrhage, as has been reported in some thromboembolic models [26].

In edaravone treatment, we followed the previous protocol using three injections due to its short half-life (T_1/2_  = 5.4 min) [12]. In animals that received edaravone (3 mg/kg, IP, <10 min and 1 and 2 h after tHI), the mortality rate at 24 h recovery dropped to 6.3%, and the infarct size was reduced to 22.2% (p<0.001 compared to vehicle-treated mice). These results support the notion that edaravone is an effective therapeutics of ischemic stroke on its own [27], [28], [29].

Moreover, when edaravone was added to the tPA-treatment at 0.5 h (E + tPA 0.5 h), the mortality rate also dropped to 9.1%, and the mean infarct volume was further reduced to 6.4%, which is significantly smaller than those receiving singular tPA (p = 0.017) or edaravone (p<0.001) treatment (Fig. 3I–K). When edaravone was added to tPA-treatment at 1 h (E + tPA 1 h), the mortality rate was similar to those received tPA alone (27.7%), but the infarct size showed a trend of further reduction (15.5%, p = 0.40 compared to the tPA-treatment alone). Finally, when edaravone was added to tPA-treatment at 4 h (E + tPA 4 h), both mortality rate (26.6%) and the mean infarct volume (30.5%) were larger than those receiving tPA alone (Fig. 3 I–K).

Together, these results suggest that edaravone may increase the benefits of early, but not late, tPA therapy in the tHI thrombotic stroke model.

### tPA-edaravone therapy improves post-stroke survival and neurological functions

Based on the results in 24 h mortality rate and infarct size, the combination of edaravone and tPA administration at 0.5 h recovery (hereafter referred to as E + tPA therapy) provided the maximal protection. Hence, we further compared the acute tPA-edaravone treatment with vehicle-controls in a series of secondary end-point analysis.

**Figure 4:**
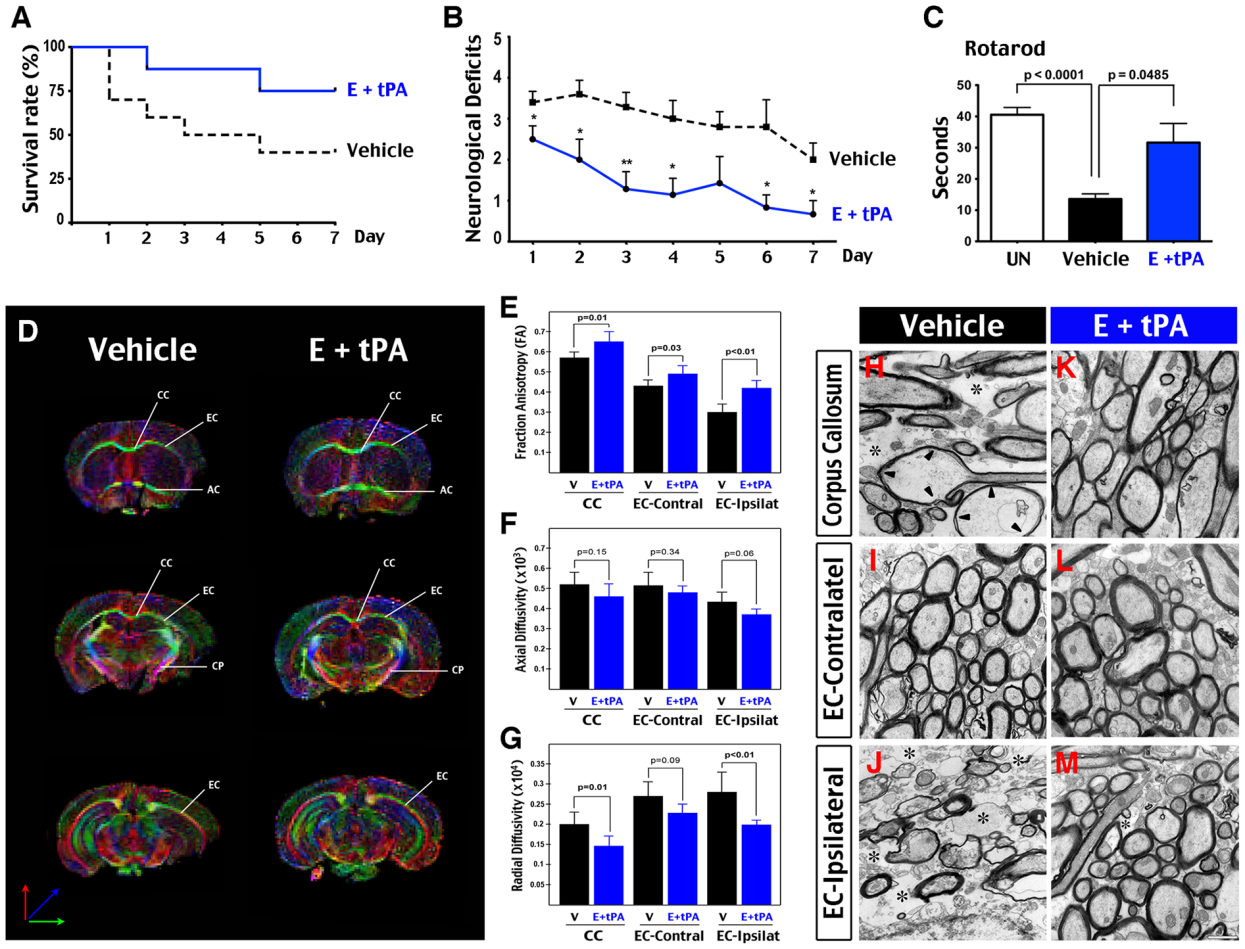
Improvement of long-term functional outcome and protection of white-matter from tHI injury by the dual tPA-edaravone therapy. (**A-B**) Survival rate (A) and long-term functional outcome (neurological deficits score, B) were monitored for 7 days following tHI in vehicle-treated and dual tPA-edaravone-treated animals (E + tPA; tPA was administered at 0.5 h). E + tPA-treated mice showed a higher survival rate (75%, n = 8) than the vehicle group (40%, n = 10), and conferred fewer neurological deficits. *, p<0.05; **, p<0.01 compared to vehicle control group by t-test. (**C**) Motor behavior was examined by rotarod testing at 7-day after tHI. The latency on rotarod (seconds) in vehicle-treated animals (n = 4) was significantly shorter than that in E + tPA-treated animals (n = 6), which is similar to the response in unchallenged (UN) animals (n = 9). (**D**) Representative coronal images of directionally encoded color (DEC) map of the brains from vehicle-treated and E + tPA-treated mice at 24 hours recovery. The directions of color-encoded water diffusion along the x, y, and z axis were indicated. (**E–G**) Comparison of the fractional anisotropy (FA), axial/longitudinal diffusivity, and radial/horizontal diffusivity in the midline corpus callosum (CC), and contralateral and ipsilateral external capsule (EC) between the vehicle-treated (n = 6) and E + tPA-treated mice (n = 4). (**H–M**) Electron micrographs of the indicated axonal tracts following tHI insults in vehicle- or E + tPA-treatment. The CC of vehicle-treated, but not E + tPA-treated animals exhibited numerous empty space (asterisks in H) and myelinated axons filled with large vacuoles (arrowheads in H). The contralateral EC in both groups showed minimal ultrastructural abnormality (I, L). The ipsilateral EC in vehicle-treated animals showed near-complete degradation of the cytoplasm in the vast majority of cell bodies and axons (asterisks in J). In contrast, the ipsilateral EC in E + tPA-treated animals showed minimal ultrastructural pathology, and smaller inter-axonal empty space (asterisk in M). Scale bar: 1 μm in H–M.

First, we compared the survival and neurological deficits between controls (n = 10) and E + tPA treatment (n = 8) over 7 days after the tHI insult. At the end of the 7 d period, only 40% of control mice survived, in contrast to the 75%-survival rate in E + tPA-treated animals (Fig. 4A). Further, the control mice showed greater neurological deficits than the E + tPA-treated animals throughout the survival period (Fig. 4B, shown are the mean and standard errors; *: p<0.05; **: p<0.01 by *t*-test). At the end of the 7 d period, vehicle-treated mice showed a shorter latency on rotarods than untouched controls and E + tPA-treated animals (Fig. 4C).

### tPA-edaravone therapy reduces ischemic white-matter injury

White matter (WM) is another important therapeutic target in acute ischemic stroke [30]. While diffusion tensor imaging (DTI) readily detects alterations of WM signal in acute stroke patients, it is difficult to interpret these results due to lack of DTI-neuropathology correlation data. Thus, we used DTI-electron microscopy (EM) correlation to test whether or not acute E + tPA therapy confers WM protection and, if so, which DTI parameters are reliable indicators of structural WM damage in this thrombotic stroke model.

Vehicle- and E + tPA-treated mice were sacrificed at 24 h recovery for sequential ex-vivo DTI and EM analysis, as described [14]. The regions-of-interest (ROI) were drawn in the midline corpus callosum (CC) and the external capsule (EC) in directionally encoded color (DEC) maps to derive DTI parameters, including fractional anisotropy (FA, an index of overall directionality), axial diffusivity (an index of water diffusion along the trajectory of axons), and radial diffusivity (an index of water diffusion perpendicular to the trajectory of axons) between vehicle (n = 6) and E + tPA-treated mice (n = 4) (Fig. 4D–G). Subsequently we used EM to examine the corresponding axonal bundles (Fig. 4H–M).

On DTI analysis, the ipsilateral EC in vehicle-treated animals showed 27% reduction of FA and 45% increase in radial diffusivity compared with E + tPA-treated animals (p<0.01 for both) (Fig. 4E, G). On EM, the ipsilateral EC in vehicle-treated mice showed severe axonal loss and large expansion of interaxonal space (asterisk in Fig. 4J), while the ipsilateral EC in E + tPA-treated mice showed normal ultrastructure and small interaxonal space (asterisk in Fig. 4M).

In the midline corpus callosum, vehicle-treated animals showed 12% reduction of the FA value and 38% increased in radial diffusivity than E + tPA-treated mice (p = 0.01 for both; Fig. 4E, G), which are less than the differences between vehicle-controls and E + tPA-treated animals in the ipsilateral EC. Similarly, vehicle-controls showed more modest pathological changes on EM, including swollen axons with near-complete degradation of axoplasmal contents (arrows in Fig. 4H). In contrast, such alterations were not detected in E + tPA-treated animals (Fig. 4K).

In the contralateral EC, no clear ultrastructural pathology was detected in mice receiving either treatment (Fig. 4I, L). Accordingly, although vehicle-treated animals showed substantial reduction of the FA value (p = 0.03), they failed to show significant increase in radial diffusivity, when compared to E + tPA-treated animals (p = 0.09) (Fig. 4E, G).

These results suggest that acute tPA-edaravone therapy protects WM in the tHI stroke model. Further, concurrent large FA-reduction and significant increase in radial diffusivity is a better indicator of ischemic WM injury than the decline in FA or axial diffusivity alone.

### tPA-edaravone therapy improves reperfusion and blocks multiple pathogenic mechanisms

Finally, we compared the effects of vehicle and tPA-edaravone treatment (E + tPA) on several pathogenic processes that were implicated in the permanent HI stroke mode [9].

**Figure 5:**
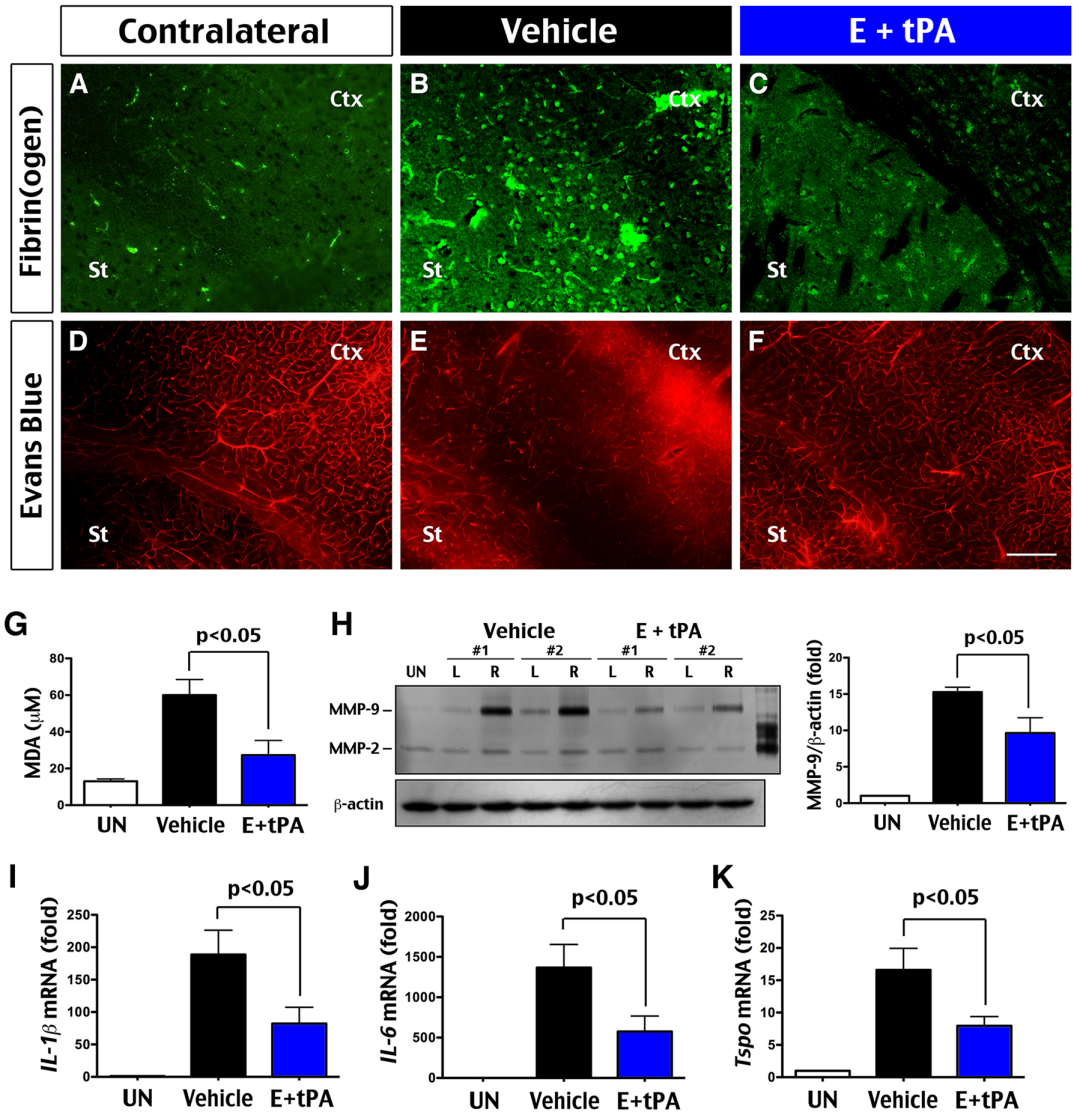
Mechanistic correlates of the dual tPA-edaravone therapy of tHI injury. (**A–F**) Immunostaining of fibrinogen (A–C) and fluorescent images of the Evans Blue dye (D–F) at 4 h after tHI injury in the striatum (St) and cerebral cortex (Ctx) of the ipsilateral hemisphere of vehicle- and E + tPA-treated animals, and the contralateral hemisphere of mice receiving either treatment. This analysis showed that the E + tPA treatment decreased fibrin deposits, perfusion deficits, and extravasation of the Evans Blue dye. (**G**) Quantification of malondialdehyde (MDA) at 24 h post-tHI showed significant reduction of lipid peroxidation by the E + tPA treatment. (**H**) MMP zymography at 24 h post-tHI and quantification showed significant reduction of MMP-9 activity by the E + tPA treatment (n = 4 for each). Equal loading of brain extracts was verified by immunoblot detection of β-actin. L: contralateral (left) hemisphere; R: The ipsilateral (right) hemisphere. (**I–K**) Real time RT-PCR showed that the E + tPA treatment significantly decreased tHI-induced IL-1β, IL-6, and Tspo transcripts at 24 h recovery (n = 4 each).

First, we compared the effects on cerebral reperfusion after tHI insults. Mice receiving vehicle or tPA-edaravone therapy were subjected to sequential transcardial perfusion of saline and fixatives at 4 h post-tHI for immunostaining of fibrin(ogen) (n = 4 for each). This analysis detected numerous punctate fibrin deposits in the ipsilateral hemisphere of vehicle-treated mice, including the striatum (St) and the cerebral cortex (Ctx), but not in the contralateral side or in E + tPA-treated animals (Fig. 5A–C). Similarly, in tHI-injured mice receiving IP injection of Evans Blue dye at 10 min before sacrifice, areas of low vascular perfusion and dye extravasation were found in the ipsilateral hemisphere of vehicle-treated mice, but not in the contralateral side or in E + tPA-treated mice (Fig. 5D–F, n = 4 for each). These results suggest that early tPA-edaravone therapy improves vascular reperfusion after tHI insults.

Next we compared the effects on several biochemical pathogenic events. By 24 h post-tHI, tPA-edaravone-treated animals showed less malondialdehyde (MDA, a marker of lipid peroxidation; [31]) than vehicle-treated mice in the ipsilateral hemisphere (Fig. 5G, p<0.05, n = 4–5 for each). Similarly, the induction of matrix metalloproteinase-9 (MMP-9, a critical mediator of BBB damage [18]) in the ipsilateral hemisphere was attenuated by combined tPA-edaravone treatment (Fig. 5H, n = 4 for each group). The tPA-edaravone therapy also reduced the rise of inflammatory cytokine transcripts, including IL-1β, IL-6, and peripheral benzodiazepine receptor (TSPO, a marker of activated microglia [32]) in the ipsilateral hemisphere (Fig. 5I–K, p<0.05, n = 4 for each).

Together, these results provided mechanistic correlates of the tPA-edaravone-mediated protection in experimental thrombotic stroke.

## Discussion

Reperfusion of ischemic brain by thrombolysis is a top priority in acute stroke therapy, but incomplete microcirculatory reperfusion may occur despite recanalization of large, primarily occluded arteries [33]. Experimental models that mimic endogenous thrombosis are useful tools to unravel the mechanisms of reperfusion deficits towards improved thrombolytic therapies. In this study, we investigated: (a) whether transient carotid artery occlusion under hypoxia provides a tPA-responsive thrombotic stroke model, and (b) whether post-tHI administration of tPA and edaravone has additive or synergistic effects. Our results support both hypotheses and have clinical implications.

### Transient hypoxia-ischemia as a simple and standardized thrombotic stroke model

It is increasingly recognized that mechanical occlusion of cerebral perfusion, such as the intraluminal suture MCAO model, has different pathophysiology from clinical ischemic stroke. This disparity may have contributed to the difficulty in translating neuroprotective therapies into clinical practice to date [6]. Yet, current thromboembolic stroke models also have drawbacks in technical complexity or low reproducibility [34]. Hence, additional thromboembolic models that respond to real-world therapies (e.g. tPA-thrombolysis) are useful for preclinical stroke research. In the present study, we show that as brief as 30 min occlusion of the unilateral common carotid artery in a hypoxic atmosphere (7.5% oxygen) is adequate to induce thrombosis and infarction in young adult C57BL/6 mice. This new thrombotic stroke model has several unique advantages for preclinical research.

First, thrombus in this new model is formed *in-situ* by endogenous components without the need for introducing exogenous chemicals or *ex-vivo* pre-formed emboli. The triggers of thrombosis in this model – decreased cerebral blood flow and hypoxia – are also physiologically relevant to clinical stroke. Because differences in clot formation influence the responsiveness to tPA [8], the tHI model may capture unique properties of endogenous thrombosis and broaden the repertoire of preclinical stroke research.

Second, the tHI stroke model responds impressively to acute tPA-thrombolysis, but less so to delayed treatment, similar to the situation in patients. We show that when tPA (10 mg/kg) is given at 30 min after the tHI insult, it significantly reduces both mortality rate and infarct size. Yet, when administered at 1 h, it reduces infarct size but not the mortality rate, and when applied at 4 h recovery, it only reduces the mean infarct size modestly. These temporal responses imitate the clinical experience of tPA-thrombolysis (beneficial if administered in <4.5 h of stroke onset), and provide an experimental system to extend its therapeutic window. However, it is noted that the therapeutic window of tPA-thrombolysis in the tHI model is shorter than that in patients. The disparity is likely caused by more intense and widespread thrombosis in the experimental model.

Third, the tHI model is simple and can be standardized across different laboratories. The surgical procedure in the tHI model (ligation of the unilateral common carotid artery) does not require dexterous skill, compared with the intraluminal suture MCAO model, and is performed under direct visualization. The other experimental conditions, including the severity and duration of hypoxia, are all objective and controllable, hence decreasing procedural variations. The ability to compare one another's findings with a relatively uniform stroke model will increase the rigor of preclinical research.

**Figure 6:**
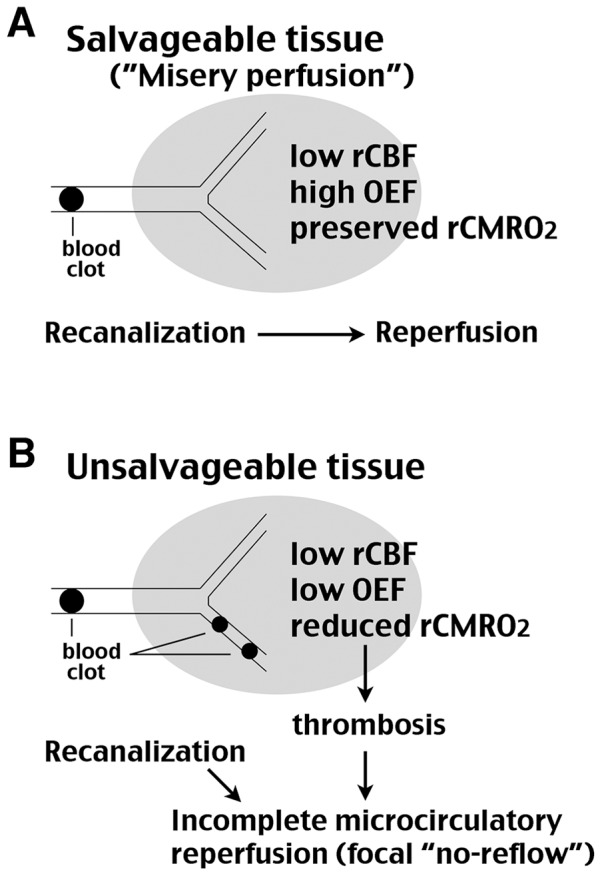
Hypothesis on how HI-induced thrombosis induces reperfusion deficits in stroke. (**A**) Reperfusion is coupled to recanalization in salvageable ischemic tissue where a near-normal regional metabolic rate of oxygen (rCMRO_2_) is maintained due to an elevated oxygen extraction fraction (OEF) in response to reduced regional cerebral blood flow (rCBF). (**B**) In contrast, dual reduction of rCBF and rCMRO_2_ inside unsalvageable ischemic tissue triggers thrombosis and disrupts the recanalization-reperfusion coupling, leading to focal “no-reflow” or “incomplete microcirculatory reperfusion” as described in the literature [3], [33].

Last but not least, the tHI model may shed insights into the mechanisms of incomplete microcirculatory reperfusion after thrombolysis (Fig. 6). Imaging studies indicate that the viable ischemic area has a higher oxygen extraction fraction (OEF) to compensate for reduced CBF and maintain a near-normal regional cerebral metabolic rate of oxygen (rCMRO_2_), a condition also called “misery perfusion” (Fig. 6A) [35]. In contrast, the unsalvageable ischemic tissues have the hallmark of simultaneous reduction of OEF and CBF, and thus a diminished rCMRO_2_ (Fig. 6B). Because dual reduction of CBF and rCMRO_2_ imposes an ischemic-hypoxic insult similar to the HI model, accordingly, non-viable ischemic tissues may develop secondary thrombosis that leads to reperfusion deficits after thrombolysis. Thus, the tHI model may assist better understanding the mechanisms of secondary thrombosis towards improved thrombolytic therapies in patients.

### Edaravone as an adjunctive or prophylactic therapy of ischemic stroke

Edaravone has been approved for treating acute cerebral ischemia in Japan since 2001, but its worldwide clinical development is still in its infancy [29], [36]. Edaravone is a lipophilic free radical scavenger that neutralizes multiple reactive oxygen species and protects both neurons and endothelial cells in the neurovascular unit [24], [37]. It also exerts anti-apoptosis and anti-inflammation actions that contribute to cytoprotection in ischemic stroke [27], [28], [38]. Importantly, edaravone readily crosses the BBB, which is a major advantage for clinical use [10]. A recent open-label clinical study showed that the application of edaravone during tPA infusion enhances early recanalization in ischemic stroke patients [23]. This finding suggests synergistic benefits of combined tPA-edaravone therapy, but this hypothesis is yet to be tested in preclinical studies and double-blind clinical trials.

To this end, we examined the effects of edaravone and dual tPA-edaravone therapy in the tHI-induced thrombotic stroke model. Our results show that post-tHI application of edaravone by itself reduces both mortality rate and infarct size, consistent with its reported beneficial effects in ischemic stroke [37]. Further, when edaravone is combined with early tPA administration (at 0.5 h recovery), the infarct size is smaller than those receiving tPA or edaravone alone (p<0.05), thus fulfilling the criteria for synergy. The acute tPA-edaravone treatment also improves vascular reperfusion and provides better survival and behavioral outcomes. Yet, when edaravone is combined with tPA administration after 1 h recovery, the additive benefits either fall short of statistical significance or disappear, similar to the findings in a rabbit small clot embolic model [13]. The lack of synergy between edaravone and tPA in the rabbit emboli model was attributed to a “ceiling effect” due to the standard high dose of recombinant tPA (Activase) used in non-human animal species [39]. This scenario is supported by a recent study demonstrating that the “human” dose of Activase (0.9 mg/kg) is already effective in a rat embolic stroke model, albeit to a lesser degree than the standard high dose (10 mg/kg) [40]. Because our study also used the 10 mg/kg dose of recombinant tPA, it is possible that our results may under-estimate the window for synergistic or additive benefits between tPA and edaravone. Future studies with various doses of tPA and edaravone combination are warranted to clarify this issue.

Finally, although the present results do not confirm the report of edaravone extending the therapeutic window of tPA thrombolysis [41], there are important differences in research design. In the previous study, edaravone was applied during MCA occlusion, while our study uses post-tHI intervention. Hence, better outcomes with edaravone co-treatment in the previous study may signify its prophylaxis benefits against cerebral ischemia. In this regard, it is worth noting that the insult in the tHI model (transient cerebral ischemia-hypoxia) is similar to those experienced by individuals with chronic carotid stenosis undergoing major surgery. Because these individuals have a greater risk for post-operative stroke or cognitive impairment [42], our results suggest that they may benefit from prophylactic edaravone treatment either before or soon after surgery.

In summary, this study introduces a simple and tPA-responsive thrombotic stroke model. We also show for the first time synergy between edaravone and tPA in reducing the infarct size. Given these results, we suggest that further studies of the combinational tPA-edaravone therapy, in accordance with recommendations by the Stroke Therapy Academic Industry Roundtable [43], merit consideration to test its clinical potential.
